# Linkage Analysis and Association Mapping QTL Detection Models for Hybrids Between Multiparental Populations from Two Heterotic Groups: Application to Biomass Production in Maize (*Zea mays* L.)

**DOI:** 10.1534/g3.117.300121

**Published:** 2017-09-28

**Authors:** Héloïse Giraud, Cyril Bauland, Matthieu Falque, Delphine Madur, Valérie Combes, Philippe Jamin, Cécile Monteil, Jacques Laborde, Carine Palaffre, Antoine Gaillard, Philippe Blanchard, Alain Charcosset, Laurence Moreau

**Affiliations:** * Génétique Quantitative et Évolution-Le Moulon, Institut National de la Recherche Agronomique, Université Paris-Sud, Centre Nationnal de la Recherche Scientifique, AgroParisTech, Université Paris-Saclay, F-91190 Gif-sur-Yvette, France; † Unité Expérimentale 0394 Saint-Martin-de-Hinx Maïs, Institut National de la Recherche Agronomique, F-40390 Saint-Martin-de-Hinx, France; ‡ Maïsadour Semences SA, F-40001 Mont de Marsan Cedex, France; § Euralis Semences, Domaine de Sandreau, F-31700 Mondonville, France

**Keywords:** hybrids, QTL detection, additivity, dominance, silage maize, multiparental populations, MPP

## Abstract

Identification of quantitative trait loci (QTL) involved in the variation of hybrid value is of key importance for cross-pollinated species such as maize (*Zea mays* L.). In a companion paper, we illustrated a new QTL mapping population design involving a factorial mating between two multiparental segregating populations. Six biparental line populations were developed from four founder lines in the Dent and Flint heterotic groups. They were crossed to produce 951 hybrids and evaluated for silage performances. Previously, a linkage analysis (LA) model that assumes each founder line carries a different allele was used to detect QTL involved in General and Specific Combining Abilities (GCA and SCA, respectively) of hybrid value. This previously introduced model requires the estimation of numerous effects per locus, potentially affecting QTL detection power. Using the same design, we compared this “Founder alleles” model to two more parsimonious models, which assume that (i) identity in state at SNP alleles from the same heterotic group implies identity by descent (IBD) at linked QTL (“SNP within-group” model) or (ii) identity in state implies IBD, regardless of population origin of the alleles (“Hybrid genotype” model). This last model assumes biallelic QTL with equal effects in each group. It detected more QTL on average than the two other models but explained lower percentages of variance. The “SNP within-group” model appeared to be a good compromise between the two other models. These results confirm the divergence between the Dent and Flint groups. They also illustrate the need to adapt the QTL detection model to the complexity of the allelic variation, which depends on the trait, the QTL, and the divergence between the heterotic groups.

Multiparental populations (MPP) have proved to be efficient for detecting loci involved in the variation of quantitative traits. Compared to biparental populations, they enable the exploration of more allelic diversity and improve the power and accuracy of QTL detection. Contrary to genome-wide association mapping based on panels of inbred lines, MPP designs composed of several biparental population families have a clear population structure. Controlling it in statistical analyses helps to prevent the risk of false positives due to associations between loci that are not physically linked. Such designs also permit alleles to be traced from the founder “parental lines” to the segregating populations, allowing the implementation of several detection models. The first joint analyses of several segregation populations considered that each founder line carried a different allele. In the case of Maize (*Zea mays* L.), this joint LA model led to the detection of allelic series for several traits of interest, *i.e.*, at least three significantly different parental allele effects (Rebaï *et al.* 1997; Blanc *et al.* 2006; Buckler *et al.* 2009; Giraud *et al.* 2014). However, when the number of parents is high, this model may be overparametrized, especially when founder lines are related. Such founder relationships are a common feature in breeding programs, and lead to local IBD. In such a case, dense SNP genotyping of the founder parental lines enables application of a genome-wide association study model to reduce the number of parameters in the QTL detection and potentially increase the mapping resolution. This approach (often referred to as LDLA mapping) was first proposed by Yu *et al.* (2008) for NAM designs and was applied efficiently in the US-NAM design to fine-map QTL for several traits up to the gene level (Kump *et al.* 2011; Tian *et al.* 2011). It makes the implicit assumption that the QTL are biallelic and that the allelic effects do not depend on the genetic background. Several studies evaluated the properties of these different models by simulations (Li *et al.* 2016) or empirically (Kump *et al.* 2011; Tian *et al.* 2011; Bardol *et al.* 2013; Giraud *et al.* 2014; Garin *et al.* 2017) and consistently found that the models were complementary, with efficiencies depending on the trait, the design, and the QTL considered.

In cross-pollinated species, heterosis is important for traits related to yield. Hybrids of agronomical interest are derived from crosses between unrelated individuals belonging to complementary genetic groups (heterotic groups). In this context, the value of a hybrid can be decomposed as the sum of the additive value of each of its parents (their GCA) and the interaction between its two parents (the SCA of the two parents) (Sprague and Tatum 1942). Identifying loci involved in these two components is of interest in hybrid breeding. Most MPP designs evaluated so far for intergroup hybrid value have involved crosses of segregating individuals to a common unrelated line (“tester”). The variation of hybrid performances in such designs only reflects the allelic variation in the group of parental founder lines of the MPP. Also, as the GCA allelic effects are confounded with their SCA with the tester alleles, additive and dominant QTL effects cannot be distinguished and part of the variation of the MPP design can be masked by dominant tester alleles.

In a companion paper, we proposed a new design to extend multiparental QTL detection designs to factorials between two heterotic groups (Giraud *et al.* 2017). This design relies on two multiparental maize populations corresponding to the two most important heterotic groups used in Europe (Dent and Flint). Within each heterotic group, a multiparental mapping population was developed from six connected biparental populations of segregating lines, issued from four founder lines. Instead of using “testers” (a common unrelated inbred line crossed to all experimental lines) to evaluate their hybrid values, segregating lines were crossed according to an incomplete factorial design to produce Dent-Flint hybrids. These hybrids were evaluated for biomass production. A first QTL detection was carried out to identify loci involved in the GCA and SCA components of hybrid value. This analysis was conducted considering allelic effects transmitted from each founder line. This model is an extension of joint LA to factorial models. It proved very encouraging to detect jointly GCA QTL in the two heterotic groups and test for SCA effects. The results suggested that most of the GCA QTL were specific to each group and no QTL with significant SCA effects at the genome level were found. This analysis raises issues regarding the number of parameters that need to be estimated at each QTL. With four founder lines per group, three independent GCA effects per group and nine independent SCA effects were estimated, leading to a total of 15 effects per locus. This might have negatively impacted the power of QTL detection. Therefore, the question of the potential of allele clustering appears to be particularly important in this context to (i) restrict the number of parameters to be estimated and (ii) test the consistency of allelic effects in the two groups. The first point is particularly critical for dominance effects in designs that would involve a high number of parents in each group.

The objective of this study is therefore to propose alternative QTL detection models adapted to the analysis of factorials between segregating populations, and test their efficiency in the factorial design proposed by Giraud *et al.* (2017). Comparison of the different QTL models in such design can provide insight into the importance of allelic series and the divergence between the Dent and Flint groups.

## Materials and Methods

### Genetic material

**Figure 1:**
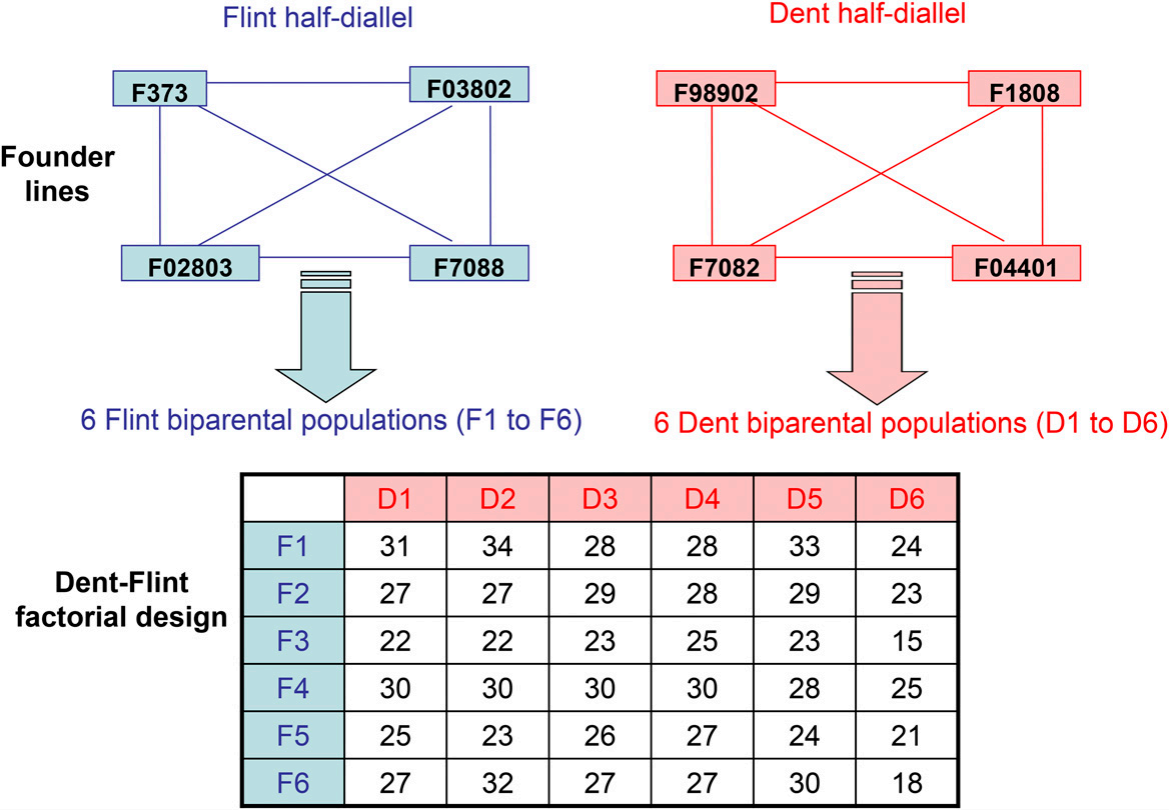
Schematic representation of the experimental design. The table shows the number of Dent-Flint hybrids retained for QTL detection for each of the 36 Dent-Flint combinations of biparental populations. QTL, quantitative trait loci.

The experimental material consists of a Dent-Flint factorial between segregating lines used in the companion paper by Giraud *et al.* (2017). Four founder inbred lines were chosen within each heterotic group (Dent and Flint): one for its digestibility and the others for their yield potential. In each group, all crosses between pairs of founder lines were made to produce six biparental populations (Figure 1). In total, 931 Dent lines and 913 Flint lines were obtained by doubled haploidization and five to six generations of selfing, respectively. From these parental lines, 863 Dent lines and 879 Flint lines were crossed in an incomplete factorial design in order to produce 1044 experimental Dent-Flint hybrids. Each biparental population of one group was crossed with all the biparental populations of the other group, with the objective of balancing their contribution. The majority of lines (699 in the Dent and 732 in the Flint) contributed to only one hybrid, but some lines contributed twice (163 in the Dent group and 146 in the Flint group). Only two lines contributed to three or four hybrids. All founder lines of one group were crossed with the founder lines of the other group to create 16 hybrids that were used as checks.

### Genotyping data

The founder lines were genotyped with an Illumina 50 K SNP array (Ganal *et al.* 2011). The founder lines and the parental lines were genotyped for a subset of 18,480 SNPs with an Affymetrix array designed by Limagrain. For the analyses, we considered the Affymetrix genotyping data for the founder lines, and when possible replaced missing data by the genotypes obtained with the 50 K SNP array. To avoid ascertainment bias, we only considered the PANZEA markers (Ganal *et al.* 2011) that were polymorphic among the founder lines. We restricted the analyses to loci that had <20% missing values within the Dent and Flint sets of parental lines. Markers with >5% of heterozygosity among the Dent parental lines or in total, or >10% of heterozygosity among the Flint parental lines (issued from single seed descent and therefore with some expected residual heterozygosity) were discarded. Markers with a Minor Allele Frequency (MAF) <5% were discarded. After applying the above-mentioned criteria, 9643 markers were retained for further analyses.

Genotyping failed for nine inbred lines. Genotype consistency between founder lines and parental lines was checked and off-type lines were excluded, as well as inbred lines showing a high level of heterozygosity (>25 and 10% for the Flint and the Dent lines, respectively). In total, 875 Dent lines and 883 Flint lines were retained for further analyses. This data can be found in the supplemental material of Giraud *et al.* (2017).

These 1758 inbred lines were used to build 12 genetic maps, one for each of the 12 biparental populations as well as one Dent-Flint consensus map, established following the approach described in Giraud *et al.* (2014). Segregation distortion was tested within each population and markers with unexpected segregation were discarded prior to map building. Most allele frequencies ranged from 0.4 to 0.6 except for a few chromosome segments in some populations (for instance on chromosome 2 for the population D6), suggesting no strong involuntary selection during line development. On average, distances between consecutive map positions were <2 cM but large gaps (>10 cM) were observed (for instance on chromosome 4 for the population F2), corresponding to chromosome regions where the two founder lines of the population were IBD (Supplemental Material, Table S1 in File S5). The Dent-Flint consensus map comprised 9548 markers that were polymorphic in at least one Dent or one Flint population. This map has a total length of 1578.6 cM and contains 5216 unique positions (*cf*. File S1 for the consensus and individual population maps). Missing genotypes of parental lines were imputed with Beagle v3.0. (Browning and Browning 2009). As Dent lines were doubled haploids, heterozygous loci for Dent lines were considered as missing data and were imputed considering that only homozygous genotypes were possible. Imputations were performed within each population with the founder lines included. Phasing (for the Flint lines and the founder lines) and missing genotype imputation were done at the same time.

### Field trial design and estimation of least squares-means (ls-means)

Hybrids were evaluated in eight different environments (four locations in 2013 and four in 2014) in the North of France and in Germany. Trials were conducted following common agricultural practices of the region. Four traits were measured: silage yield (DMY in tons of dry matter per ha), dry matter content at harvest (DMC in % of fresh weight), plant height (six environments) (PH in centimeters) and female flowering (DtSILK in days after January the first, scored as the date at which 50% of the plants of the elementary plot exhibited stigmas, referred to as “silks” in maize). The field experiments consisted of 1088 experimental units, each a field plot of two 5 m-long rows. The experimental design was laid out as an augmented p-rep design (Williams *et al.* 2011). The hybrids between founder lines and ∼17% of the experimental hybrids were evaluated twice per environment, whereas most of the hybrids between the parental inbred lines were evaluated only once. Trials were laid out in 68 incomplete blocks consisting of 16 plots each, with five to six plots used for repeated genotypes (experimental hybrids and checks). In total, 1044 hybrids were evaluated over the whole experimental design. After removing outlier observations (at the phenotypic and genotypic level), 951 hybrids were considered for QTL detection (950 for PH and DMY), involving 822 and 802 parental lines in the Flint and Dent group, respectively. The hybrids retained for the QTL detection belonged to all of the 36 hybrid populations corresponding to the crosses between the six Flint and the six Dent line populations [figure 1 and table 1 of Giraud *et al.* (2017)].

QTL detection was based on the ls-means of each hybrid over the environments. For DMY, data from one of eight environments were excluded as they were not correlated with the other environments. For each trait, we first corrected single-plot values by spatial effects obtained by analyzing jointly all the field trials and considering for each trial the best spatial model (row-column model or block model). Ls-means of hybrids were derived from the following model: $Y_{hlxyz}^{c}=\text{μ}+\lambda_{l}+H_{h}+E_{hlxyz},$ where $Y_{hlxyz}^{c}$ is the performance of the hybrid h located at position x, y, and block z in the environment l, corrected for the spatial field effects, μ is the intercept, ${\text{λ}}_{l}$ is the fixed effect of the environment l, and $H_{h}$ is the hybrid genetic effect considered as fixed. $E_{hlxyz}$ is the residual of the model $E_{hlxyz}\;\to N\left(0,\;{\text{σ}}_{E}^{2}\right)$. Details of the models used for spatial correction are described in Giraud *et al.* (2017). The R-script used to estimate ls-means is included in the supplemental material of Giraud *et al.* (2017).

### QTL detection

**Figure 2:**
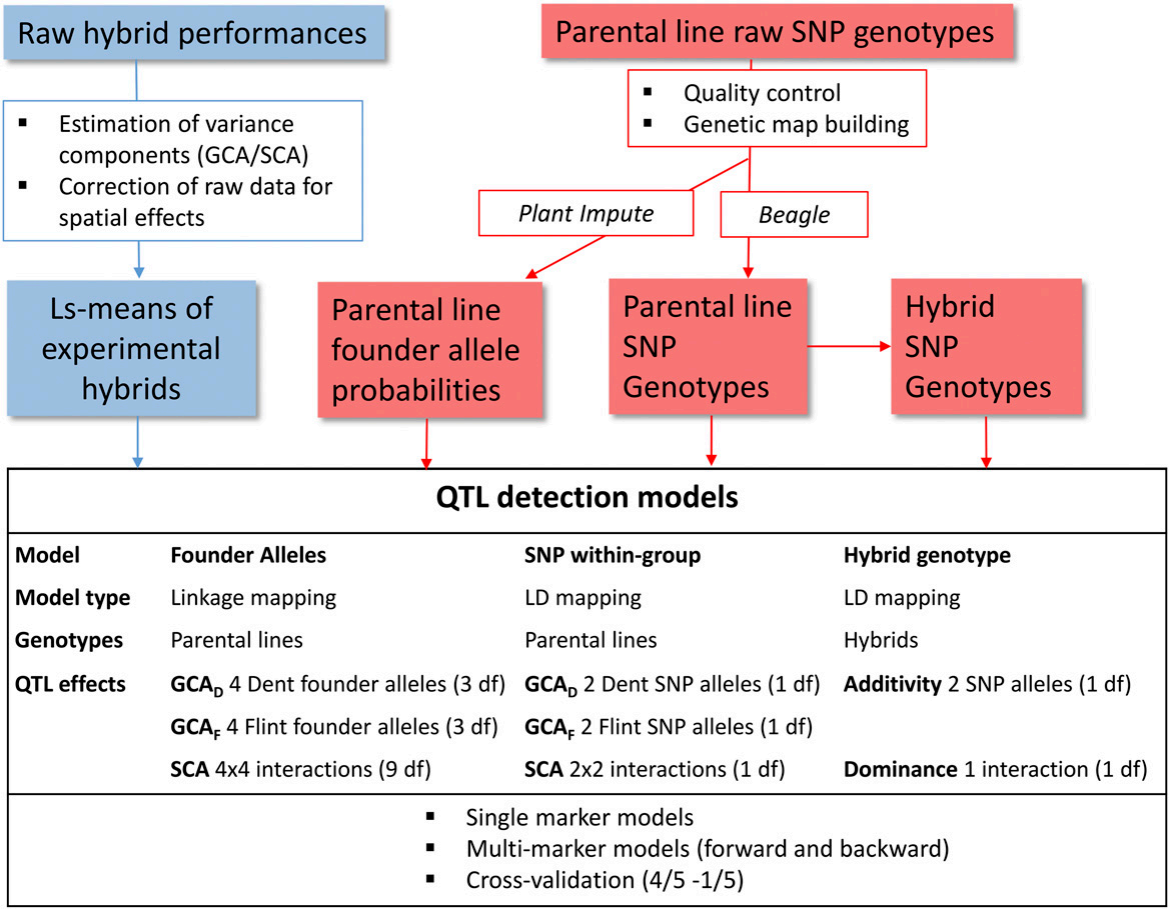
Workflow of data analysis for the phenotypic data, genotypic data and QTL detection. For each QTL model we indicated the number of d.f. corresponding to the QTL effects. Methods and results of the estimation of variance components are presented in Giraud *et al.* (2017). GCA, General Combining Ability; LD, linkage disequilibrium; ls-means, least squares-means; SCA, Specific Combining Ability; SNP, single nucleotide polymorphism; QTL, quantitative trait loci.

Three models were used for QTL detection differing by (i) the fact that they consider either the alleles transmitted by the parents of the hybrids or directly the hybrid genotypes, and (ii) the type of allele coding considered (Figure 2). The population structure of the design was taken into account for all models. Also, we included random genetic effects corresponding to the parents of the hybrids to account for the fact that some parental inbred lines were involved in several hybrids.

The Founder alleles model is the one described in Giraud *et al.* (2017). It considers the founder alleles transmitted to the hybrids and makes the assumption that each of the eight founder lines carries a different allele.

$$\begin{array}{l} y=1.\text{μ}+A.\text{α}+B.\text{β}+C.\left(\text{αβ}\right)+X_{FA\_D}.{\text{γ}}_{FA\_D}+X_{FA\_F}.{\text{θ}}_{FA\_F}+X_{FA\_DF}.{\left(\text{γθ}\right)}_{FA}+Z_{D}.u_{D}+Z_{F}.u_{F}+e \end{array}$$ (1)

where y is a (*N* × 1) vector of the ls-means of the N experimental hybrids phenotyped for the considered trait; μ is the intercept, 1 is a (*N* × 1) vector of 1. The term α (respectively β) is a (6 × 1) vector of the fixed effects of the Dent (respectively Flint) populations of origin of the Dent (Flint) parental line, (αβ is a (36 × 1) vector of the fixed interaction effects between the Dent and Flint populations of parental lines. *A*, *B*, and *C* are the corresponding design matrices. $u_{D}$ (respectively $u_{F}$) is a ($N_{D}$ × 1) [respectively ($N_{F}$ × 1)] vector of the random effects of the $N_{d}$ Dent (respectively $N_{F}$ Flint) parents, with $u_{D}∼N\left(0,I{\text{σ}}_{D}^{2}\right),$ (respectively$\;u_{F}∼N\;\left(0,I{\text{σ}}_{F}^{2}\right)$). $Z_{D}$and $Z_{F}$are the corresponding design matrices. $u_{D}$ and $u_{F}$are GCA effects not accounted for by the QTL. *e* is a (*N* × 1) vector of the residuals of the model with $e∼N\left(0,I{\text{σ}}_{e}^{2}\right).$ The QTL effect is decomposed into three terms: ${\text{γ}}_{FA\_D},$ ${\text{θ}}_{FA\_F}$, and ${\left(\text{γθ}\right)}_{FA}.$ The first term ${\text{γ}}_{FA\_D}$ (respectively ${\text{θ}}_{FA\_F}$) is the (4 × 1) vector of the allelic effects at the marker associated with each Dent (Flint) founder line. These effects correspond to the GCA effects of the QTL. For each marker, $X_{FA\_D}$ (respectively $X_{FA\_F}$) is a (*N* × 4) matrix of the probabilities that the hybrid received its Dent (respectively Flint) allele from each of the four Dent (respectively Flint) founder lines. ${\left(\text{γθ}\right)}_{FA}$ is the (16 × 1) vector of the interactions, or SCAs, between the founder alleles; $X_{FA\_DF\;}$is a (*N* × 16) matrix corresponding to the elementwise product between each column of $X_{FA\_D}$and each column of $X_{FA\_F}.$ As the sum of probabilities for each allele equals 1, this model has three d.f. for the additive effects of the founder alleles (GCAs) in each group and nine d.f. for the interaction effects (SCA). Probabilities that a hybrid received one of the four Dent (respectively Flint) founder alleles were inferred for each position of the 9548 mapped markers based on the genotypes of its parental lines at the closest informative markers. These probabilities were computed with PlantImpute (Hickey *et al.* 2015) using 10 iterations.

The SNP within-group model considers the observed alleles at SNPs received from the parental inbred lines, assuming different effects in the two heterotic groups. This model assumes that two inbred lines from the same group sharing the same allele at a given SNP are IBD at this position and transmit the same QTL allele to the hybrids.

$$\begin{array}{l} y=1.\text{μ}+A.\text{α}+B.\text{β}+C.\left(\text{αβ}\right)+X_{SNP\_D}.{\text{γ}}_{SNP\_D}+X_{SNP\_F}.{\text{θ}}_{SNP\_F}+X_{SNP\_DF}.{\left(\text{γθ}\right)}_{SNP}+Z_{D}.u_{D}+Z_{F}.u_{F}+e \end{array}$$ (2)

All effects are defined as in the Founder alleles model, except that for the QTL effects two alleles segregate in each group instead of four. The model estimates the contrast between these two allelic effects by performing a regression on the SNP minor allele frequencies. The first term ${\text{γ}}_{SNP\_D}$(respectively ${\text{θ}}_{SNP\_F}$) is the GCA effect associated with the Dent (respectively Flint) minor allele, $X_{SNP\_D}$ (respectively $X_{SNP\_F}$) is a (*N* × 1) vector of marker genotypes for the Dent (respectively Flint) parent of the hybrid, coded as 0 for homozygotes for the major allele, 1 for homozygotes for the minor allele, and 0.5 for heterozygotes (only Flint lines, see above). ${\left(\text{γθ}\right)}_{SNP}$ is the SCA effect between the minor SNP marker alleles of each group, $X_{SNP\_DF}$ is a (*N* × 1) column vector corresponding to the Hadamard product of $X_{SNP\_D}$ and $X_{SNP\_F}.$ This model has one d.f. for the GCA effect of each group and one d.f. for the SCA.

The Hybrid genotype model considers the marker genotypes of the hybrids and ignores the group origin of the alleles transmitted by the parents. It assumes that the QTL effects are the same in both heterotic groups and decomposes the QTL effect into additivity and dominance terms.

$$y=\;1.\text{μ}+A.\text{α}+B.\text{β}+C.\left(\text{αβ}\right)+X_{a}.\text{ω}+X_{d}.\text{δ}+Z_{D}.u_{D}+Z_{F}.u_{F}+e\;$$ (3)

Compared to the previous models, QTL effect is decomposed into two terms ω and δ, which are, respectively, the additive and dominance effect at the marker. $X_{a}$ is a (*N* × 1) vector coded in −1, −0.5, 0, 0.5, and 1, corresponding to the genotypes of the hybrids, inferred from the genotypes of their parental lines. $X_{a}\left(h\right)$ = −1 when the hybrid is homozygous for the major allele, 1 when it is homozygous for the minor allele, 0 when it is heterozygous, and −0.5 (respectively 0.5) when the Dent parent is homozygous for the major (respectively minor) allele and the Flint parent is heterozygous. $X_{d}$ is a (*N* × 1) vector coded in 0, 0.5, and 1. $X_{d}\left(h\right)$ equals 0 when the hybrid is homozygous, 0.5 when the Dent parent is homozygous and the Flint parent is heterozygous (note that the reverse is not possible as Dent parents are doubled haploids), and 1 when the hybrid is heterozygous. This model has one d.f. for the additive effect and one d.f. for the dominance effect.

QTL detection was performed with the package ASReml-R (Butler *et al.* 2007) of R (R Core Team, 2013) considering the level of significance of the Wald test for the QTL effects. For the SNP within-group and the Hybrid genotype models, QTL detection was performed on the 4758 mapped markers that were polymorphic (MAF > 5%) in both heterotic groups, whereas for the Founder alleles model it was performed on the 9548 mapped markers. For each model, we considered a 5% genome-wide significance threshold based on the number of effective markers (Gao *et al.* 2008). The total effect at each marker position was tested using the “group” function of the ASReml-R package (Butler *et al.* 2007). After the first initial single-marker scan along the genome, a multimarker procedure was implemented using a forward and backward marker selection process, similarly to the one presented in Giraud *et al.* (2017). Only markers with significant effects were kept in the final model. The R-scripts used to perform QTL detection are included in File S2.

From the final multilocus model, we estimated the percentage of phenotypic variance explained by the detected QTL $\left(R_{QTL}^{2}\right)$ and the percentage of within-population phenotypic variance explained by the detected QTL, $R_{QTL}^{2*}=\frac{R_{QTL}^{2}}{1-\;R_{pop}^{2}},$ where $R_{pop}^{2}$ is the percentage of variance explained by the population effects in a model without QTL. We also estimated the individual *R*^2^ of each QTL [see Giraud *et al.* (2017), for more details].

To evaluate the quality of prediction of these models, we performed a cross-validation approach following the procedure described in Giraud *et al.* (2017). Eighty percent of the data (training set) was sampled in each population and used to identify QTL, estimate the population and QTL effects, and predict the values of the hybrids on the remaining 20% (test set). Sampling was repeated 100 times. To limit computation time, for each sampling, the training set was used to test the significance of the QTL detected in the whole data set and only significant QTL were considered in the prediction model. The percentages of variance explained by the models were estimated by the squared correlation between the predicted and observed hybrid values of the test set. This procedure was conducted (i) without taking into account SCA/dominance QTL effects and (ii) taking them into account for QTL for which they were significant at a 5% individual risk level. A model including only the population effects and no QTL was also considered.

### Data availability

The consensus map and individual maps are available in File S1. File S2 contains the R-scripts used to perform QTL detection. The p-values of each marker in the single-marker scans for all traits and QTL models are included in File S3. Table S1 in File S5 contains information about the genetic maps. Table S2, Table S3, and Table S4 in File S5 present the QTL detection results of the final multimarker models. Figure S1, Figure S2, and Figure S3 in File S5 show the comparison of the QTL results obtained with the different models for DMC, DtSILK, and PH. Figure S4 in File S5 shows an overview of the QTL detected in this study. File S4 contains a description of the genetic material and the supplemental material. Pedigrees of the segregating populations, raw phenotypic data, adjusted means of hybrid performances, and genotypic data of parental lines are available as supplemental files of the companion paper (Giraud *et al.* 2017).

## Results

### Thresholds for QTL detection

The thresholds at a 5% genome-wide level used for QTL detection were determined as −log(p-value) equal to 4.53 for the Hybrid genotype model, 4.40 for the SNP within-group model, and 3.84 for the Founder alleles model. These differences reflect that, in the Founder alleles model, genotypic data at closely linked loci are highly correlated, leading to a lower threshold.

### Detection of QTL for all trait × model combinations

**Figure 3:**
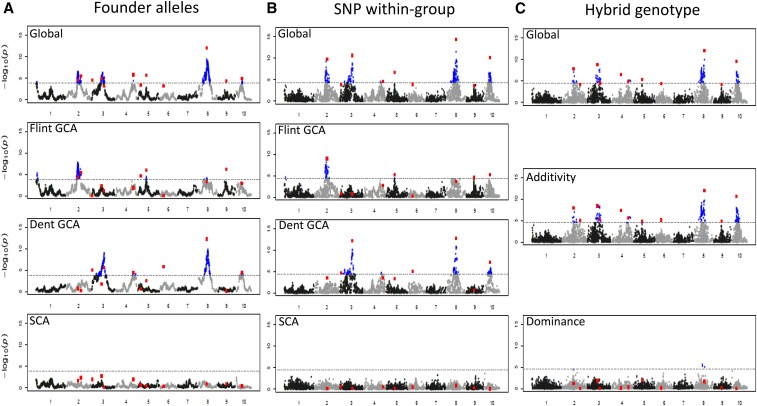
QTL detection for DMY with (A) the Founder alleles model, (B) the SNP within-group model, and (C) the Hybrid genotype model for the single-marker analysis. The chromosome number is indicated on the *x*-axis. For each model, graphics correspond to the test of the global effect (on the top) or of one component (Flint GCA, Dent GCA, and SCA effects for the Founder alleles and SNP within-group models; and additive and dominance effects for the Hybrid genotype model). The blue (black) dots correspond to positions that were above (below) the threshold in the single-marker analysis (see File S3). The red squares correspond to the −log(p-value) of the QTL that were included in the final multilocus model, with tests conditioned by the other QTL effects of the model. DMY, Dry Matter Yield; GCA, General Combining Ability; LD, linkage disequilibrium; ls-means, least squares-means; SCA, Specific Combining Ability; SNP, single nucleotide polymorphism; QTL, quantitative trait loci.

We detected QTL for all trait × model combinations. Note that results for the Founder alleles model are identical to those shown in a companion paper Giraud *et al.* (2017). As expected, we observed that the test statistics at adjacent positions were closer for the Founder alleles model than for the two others (Figure 3). For a given trait and a given QTL model, the number of detected QTL in the final multilocus models varied between 9 (DtSILK, Founder alleles model, and DMY, SNP within-group model) and 16 QTL (DtSILK and Hybrid genotype model) (Table 1). In total for the four studied traits, the SNP within-group model and the Hybrid genotype model detected more QTL (51 and 54, respectively) than the Founder alleles model, which detected in total only 42 QTL (Table 1). Nevertheless, the Founder alleles model detected more QTL for DMY. To compare the QTL detected by the different models (Figure 3 and Figure S4, Table S2, Table S3, and Table S4 in File S5), we considered that QTL detected within 10 cM of each other were identical. With this assumption, 59 QTL were specific to one model and only 16 QTL were detected with all the three models.

**Table 1:** QTL detection results with the different detection models for the different traits

				Without SCA			With SCA		
Trait	Model	Nb	*R*^2^_pop_	*R*^2^_pop+QTL_	*R*^2^_QTL_	*R*^2^*_QTL_	*R*^2^_pop+QTL_	*R*^2^_QTL_	*R*^2^*_QTL_
DMC	Founder alleles	10 (4)	32.4	60.1	27.6	40.9	63.8	32.4	47.9
	SNP within-group	12 (2)	32.4	58.3	25.5	37.7	58.9	26.1	38.6
	Hybrid genotype	14 (1)	32.4	58.6	25.6	37.9	59.0	26.2	38.8
DMY	Founder alleles	12 (5)	21.9	49.5	27.7	35.5	55.1	34.2	43.9
	SNP within-group	9 (0)	21.9	42.7	20.3	26.0	42.8	20.5	26.3
	Hybrid genotype	11 (3)	21.9	42.0	19.7	25.2	43.2	20.9	26.8
DtSILK	Founder alleles	9 (2)	15.0	46.6	31.4	36.9	51.5	36.7	43.2
	SNP within-group	15 (0)	15.0	53.1	37.3	43.9	53.3	37.6	44.3
	Hybrid genotype	16 (3)	15.0	49.9	34.1	40.2	51.3	35.6	41.9
PH	Founder alleles	11 (2)	33.8	60.0	26.6	40.2	63.0	30.7	46.4
	SNP within-group	15 (4)	33.8	58.7	24.7	37.3	60.3	26.6	40.2
	Hybrid genotype	13 (2)	33.8	54.6	20.4	30.8	55.2	21.2	32.0
Total	Founder alleles	42 (13)	25.8	54.1	28.3	38.4	58.4	33.5	45.3
	SNP within-group	51 (6)	25.8	53.2	26.9	36.2	53.8	27.7	37.4
	Hybrid genotype	54 (9)	25.8	51.3	24.9	33.5	52.2	26.0	34.9

For each method and trait, we indicate the number of QTL detected (Nb) and between brackets the number of these QTL showing significant SCA effects at a 5% individual risk level, the proportion of the phenotypic variance (*R*^2^_QTL_, in %), and of the within-population phenotypic variance (*R*^2^*_QTL_, in %) explained by the detected QTL (with and without including dominance/SCA effects in the model). The percentage of variance explained by the population effect is also indicated (*R*^2^_pop_). The total number of detected QTL and the average percentages of variance explained over the different traits are also shown. Nb, number of QTL detected; SCA, Specific Combining Ability; DMC, dry matter content; DMY, dry matter yield; DtSILK, female flowering time; PH, plant height; QTL, quantitative trait loci.

For all models, the majority of the QTL explained <5% of the variation (see Table S2, Table S3, and Table S4 in File S5). The only notable exception was a QTL detected by the three models on chromosome 10 at 44.5 cM, which explained ∼8% of the variance for DMC and 13% of the variance for DtSILK for all the detection models. Other QTL regions showed pleiotropic effects on different traits (Figure S4, Table S2, Table S3, and Table S4 in File S5).

### Decomposition of the global effect of the QTL in its different components

We tested the level of significance of GCA/SCA or additive/dominance components for all QTL that were detected (Table S2, Table S3, and Table S4 in File S5). Whatever the model considered, none of the detected QTL showed a significant dominance/SCA effect at a 5% genome-wide level. Some markers had significant dominance effects in the single-marker QTL detection scan with the Hybrid genotype model but their effects were not significant in the final multilocus model (see Figure 3 for DMY). However, some QTL were significant for SCA/dominance with an individual risk at 5%: 9 for the Hybrid genotype model, 6 for the SNP within-group model, and 13 for the Founder alleles model (Table 1 and Table S2, Table S3, and Table S4 in File S5). Among them some were significant at a 1% risk level. QTL showing SCA/dominance effects were located all over the genome. However, one region on chromosome 2, between 82.3 and 135.8 cM, stands out for presenting SCA for both DMC and DMY (Table S2, Table S3, and Table S4 in File S5).

The Founder alleles and the SNP within-group models decomposed the QTL effects into its Dent and Flint components. A majority of QTL appeared specific to one group. For the SNP within-group model, 9 QTL were significant for both GCA effects, 23 only for the Dent GCA effect, and 15 only for the Flint one (Table S3 in File S5). For the Founder alleles model, seven QTL were significant for both GCA effects, 21 only for the Dent GCA effect, and 12 only for the Flint GCA effect (Table S4 in File S5). The other QTL correspond to QTL with significant global effect but no significant individual GCA component. For QTL detected at close positions with several models, GCA/additive QTL effects of the founder lines were consistent between models (result not shown).

### Variation explained by the QTL

The detected QTL explained jointly between 19.7% (DMY, Hybrid genotype model, without dominance) and 37.6% (DtSILK, SNP within-group model, with SCA) of the total phenotypic variance, and between 26.8 and 47.1% of the within-population phenotypic variance (Table 1). The model that explained the largest fraction of the phenotypic variance was the Founder alleles model for DMY, DMC, and PH, and the SNP within-group model for DtSILK. The increase in percentage of explained phenotypic variance when taking into account dominance/SCA was low for the SNP within-group model (+0.2 for DMY to +1.9 for PH) and for the Hybrid genotype model (+0.6 for DMC to +1.5 for DtSILK), whereas it was more important for the Founder alleles model (+4.1 for PH to +6.5 for DMY) (Table 1).

Cross-validations were performed to eliminate potential bias in the *R*^2^ values of Table 1 that were computed on the data also used to estimate QTL parameters, potentially advantaging models with a high number of parameters. The highest *R*^2^ of models combining population and QTL effects were obtained with the Founder alleles model for PH and the SNP within-group model for DtSILK, and to a lesser extend DMC and DMY (Table 2). We observed a reduction of the *R*^2^ obtained by cross-validation compared to the *R*^2^ evaluated on the whole data set (*R*^2^_pop+QTL_ column of Table 1) for all the models. This reduction was stronger for the Founder alleles model. It has to be noted that, for this model, the number of QTL found to be significant when considering only four-fifths of the data were lower than the number of QTL detected using the whole data set (results not shown). The same tendency was observed, but to a lesser extent, for the other QTL models. Taking into account the dominance/SCA for the QTL for which it was significant at a 5% individual risk always had a small negative impact on the *R*^2^ of the models, especially for the Founder alleles model.

**Table 2:** Cross-validation estimates of the quality of prediction of different models (average *R*^2^ and its SD)

Model	DMC	DMY	DtSILK	PH
Population effects				
No QTL	28.4 (SD 4.18)	17.1 (SD 4.16)	10.4 (SD 2.97)	29.2 (SD 4.35)
Founder alleles				
Pop + GCA	48.2 (SD 4.48)	29.0 (SD 5.32)	32.9 (SD 4.60)	49.8 (SD 4.82)
Pop + GCA + SCA	47.4 (SD 4.58)	27.3 (SD 5.13)	32.1 (SD 4.81)	48.3 (SD 4.78)
SNP within-group				
Pop + GCA	48.8 (SD 4.33)	30.3 (SD 4.29)	39.7 (SD 5.96)	46.9 (SD 5.26)
Pop + GCA + SCA	48.6 (SD 4.48)	30.2 (SD 4.25)	39.5 (SD 6.01)	46.7 (SD 5.36)
Hybrid genotype				
Pop + Add	48.4 (SD 4.21)	28.9 (SD 4.82)	35.6 (SD 5.36)	44.9 (SD 4.96)
Pop + Add + dominance	48.2 (SD 4.23)	28.7 (SD 4.78)	35.3 (SD 5.48)	44.6 (SD 5.00)

For the different traits (DMC, DMY, DtSILK, and PH), we considered models only including population effects or models including population effects and QTL effects considering different allele codings. For these later models, for each sampling, QTL detected in the whole data set had their effects in the training set tested following a backward procedure and only the significant QTL were considered in the prediction model. Predictions were based on GCA/additive effects only or on models considering also SCA/dominance effects significant at a 5% individual risk level. DMC, dry matter content; DMY, dry matter yield; DtSILK, female flowering date; PH, plant height; QTL, quantitative trait loci; Pop, population; GCA, General Combining Ability; SCA, Specific Combining Ability; Add, additivity.

## Discussion

### Comparison of QTL detection models showed the predominance of group-specific GCA QTL

Compared to the Founder alleles model, the SNP within-group and the Hybrid genotypes models consider alleles defined at the level of SNPs (with the SNP within-group and Hybrid genotype models). These models are close to the ones used for association mapping (LD mapping), except that we used the known population structure of the design instead of a kinship matrix to control for false positives. They correspond to an extension of the models proposed for NAM designs (Yu *et al.* 2008) to the case of hybrids. The three models used for QTL detection performed differently depending on the trait and the genomic region considered. As they rely on different assumptions in terms of allelic effects, they are expected to perform differently depending on the actual distribution of QTL effects. The Hybrid genotype model considers only two d.f. per marker and is thus expected to be more powerful than the other models, which have more parameters per marker. However, it makes the strong assumptions that (i) the QTL are biallelic; (ii) they have the same effect in both heterotic groups; (iii) the marker-QTL phase is also conserved between groups; and (iv) there is no epistasis. The other models have more parameters but make fewer assumptions: (i) the effect of a given QTL and/or the marker QTL-phase depend on the heterotic group for the SNP within-group model and, in addition, (ii) each founder line has a different allele at the QTL for the Founder alleles model.

The SNP within-group model found more QTL than the Founder alleles model for all traits but DMY. This is consistent with observations by Giraud *et al.* (2014) for European NAM designs and supports the hypothesis that allelic series for yield are more complex than for other traits [see figure 3 from Giraud *et al.* (2017)].

The Hybrid genotype model detected the highest total number of QTL but it almost never explained the largest part of the genetic variance (considering direct adjustment of the data or cross-validations). The strong constraints it considers for estimating genetic effects therefore counterbalanced its advantages in terms of power. This is consistent with the detection of QTL specific to Dent or to Flint GCA by the other models. Thus, the Founder alleles and the SNP within-group models seem better adapted to QTL detection in such a design. This is in agreement with Giraud *et al.* (2014), who found different QTL in the Dent and Flint heterotic groups. The same conclusion was drawn by van Eeuwijk *et al.* (2010) when analyzing a maize factorial between two other heterotic groups for ear height and by Parisseaux and Bernardo (2004) considering intergroup hybrids obtained by crossing lines issued from a total of nine different heterotic groups. Group-specific GCA QTL may be due to actual differences in QTL allelic variability but may also result from epistatic effects. Differences in linkage disequilibrium phases between heterotic groups [as found by Lehermeier *et al.* (2014) between the Dent and Flint groups] may partly explain the lower efficiency of the Hybrid genotype model.

Whatever the model considered, we did not detect QTL with SCA/dominance effects significant at a 5% genome-wide risk level. We nevertheless detected dominance and/or SCA effects significant at a 5% individual risk level for some QTL (at a 1% individual risk level for three of them). As for the Founder alleles model, cross-validation results showed that adding SCA QTL effects to the models slightly decreased the quality of prediction of hybrid values, suggesting that these moderate QTL SCA effects may not be well estimated in training sets whatever the model considered. Reducing the number of parameters via the use of the SNP within-group or the Hybrid genotype models instead of the Founder alleles model did not help to identify QTL involved in SCA. This suggests that SCA is due to numerous small effects that are difficult to detect and/or that they involve allelic interactions (dominance) that cannot be captured by a single parameter (as assumed by models based on SNP genotypes). Another explanation might be that SCA is due to epistasis that was not included in our detection models.

Hence, results from the different models consistently show that performance of hybrids between lines from different heterotic groups is mostly affected by GCA QTL that are located at different positions in the two groups of interest. As discussed in Giraud *et al.* (2017), this result is consistent with the strong divergence between the two groups that were considered in this study and with GCA explaining 80% of hybrid variation (Reif *et al.* 2007). The small percentage of SCA variance certainly partly explains why we did not detect QTL with significant SCA at the genome level. Therefore, it would be interesting to compare the efficiency of our different models for detecting dominance/SCA effects in other experiments showing a higher contribution of SCA/dominance to the total hybrid variation. This may also be done by simulations but this is beyond the scope of this paper.

### Potential improvement of QTL detection models for a higher number of founder lines

Our results are consistent with those of Bardol *et al.* (2013) and Giraud *et al.* (2014), who also found that the model considering that each founder line carried a different allele (the Founder alleles model) was more adapted to complex traits such as yield than to simpler traits such as flowering time. As discussed in Giraud *et al.* (2017), one of the main drawbacks of this QTL detection model is that it requires the estimation of many parameters. This phenomenon is reinforced in the present hybrid design, with 35 d.f. for the combinations between the Dent and Flint populations and nine d.f. for the SCA per QTL, in addition to six d.f. for the GCA effects per QTL. This certainly explains the strong reduction of *R*^2^ observed for this model in cross-validation results compared to other models. Use of this model makes it necessary in practice to develop large segregating populations from few founder lines to get enough power and accurate QTL effect estimates. The SNP within-group model that was tested seems to be a good alternative for more complex designs, at least for traits that are expected to show “simple” allelic series (such as DtSILK). An intermediate strategy between the Founder alleles and the SNP within-group models would be to cluster the parental alleles based on their local similarities, as proposed by Leroux *et al.* (2014) and evaluated experimentally by Bardol *et al.* (2013), Giraud *et al.* (2014), and Han *et al.* (2016). Recently, Garin *et al.* (2017) reanalyzed part of the Dent NAM design of Giraud *et al.* (2014) and showed that it can be useful to mix in the same model QTL with different types of effects (parental, ancestral based on allele clustering, or biallelic). It would be interesting to adapt this strategy to factorial designs. Another possibility for more complex pedigree could be to consider QTL effects as random and use markers to compute local similarity, as done by Crepieux *et al.* (2004) on wheat inbred line data and recently by Tisné *et al.* (2015) in oil palm hybrids. van Eeuwijk *et al.* (2010) performed QTL detection in a factorial design issued from a private breeding program that was derived by crossing a large number of parental lines (but not structured in balanced families as in our design). Their analyses were based on a Bayesian model that used both molecular markers and pedigree to trace back ancestral founder alleles at QTL, assumed to be biallelic, and compute local similarity. SCA was not included in their analysis. Another improvement could be to use markers to compute kinship matrices to handle covariances between individuals due both to the population structure and the effect of unlinked QTL, as proposed by Xu (2013), and applied to QTL mapping in MAGIC populations by Wei and Xu (2016). Nevertheless, this approach needs to be adapted to factorial designs.

## Supplementary Material

Supplemental material is available online at www.g3journal.org/lookup/suppl/doi:10.1534/g3.117.300121/-/DC1.
