# Supplementary material for: Linkage Analysis and Association Mapping QTL Detection Models for Hybrids Between Multiparental Populations from Two Heterotic Groups: Application to Biomass Production in Maize (Zea mays L.)
Source: G3 (Bethesda). 2017 Sep 28;7(11):3649–57. doi: 10.1534/g3.117.300121 (PMC5677153; doi:10.1534/g3.117.300121)
Supplement: Supplementary file 5 [file 3649FileS5.docx]

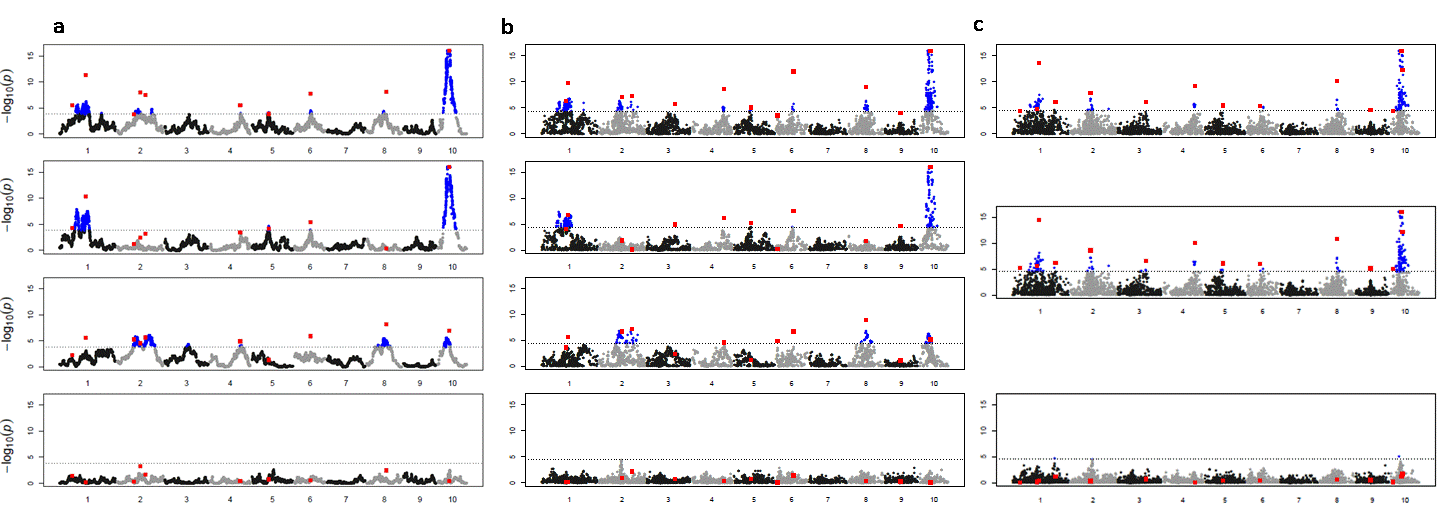


SNP within group

Additivity

SCA

Global

Global

FlintGCA

DentGCA

SCA

Dominance

DentGCA

FlintGCA

Global

Hybrid genotype

Founder Alleles

**Figure S1:** -log(p-value) curves of QTL detection for Dry Matter Content (DMC) with (a) the “Founder alleles” model, (b) the “SNP within-group” model, (c) the “Hybrid genotype” model for the single-marker analysis. The chromosome number is indicated on the X-axis. For each model, graphics correspond to the test of the global effect (on the top) or of one component (the Flint GCA, the Dent GCA and the SCA for the “Founder alleles” and “SNP within group” models and the additive and dominance effect for the “Hybrid genotype” model). The blue dots correspond to positions that were above the threshold in the single-marker analysis (see File S3). The red squares correspond to the –log(p-value) of the QTLs that were included in the final multi-locus model, with tests conditioned by the other QTL effects of the final detection model. An upper limit for the –log(pval) was fixed to 16.

**
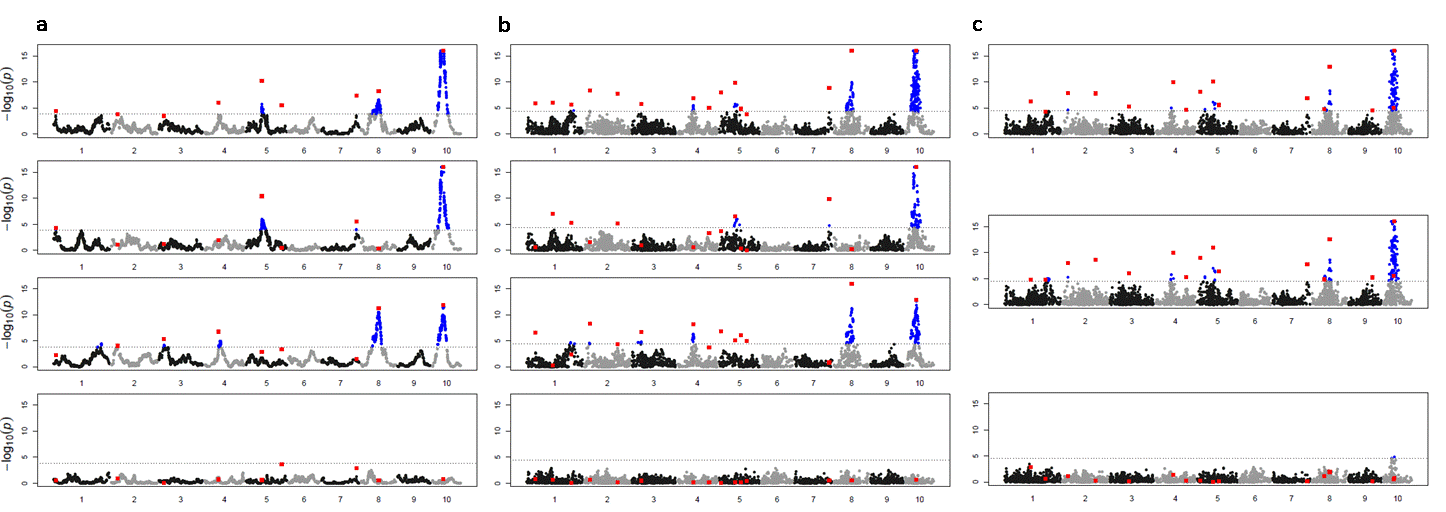
**

Dominance

Additivity

SCA

DentGCA

FlintGCA

Global

SCA

DentGCA

FlintGCA

Global

Founder Alleles

SNP within group

Hybrid genotype

Global

**Figure S2:** -log(p-value) curves of QTL detection for Female Flowering (DtSILK) with (a) the “Founder alleles” model, (b) the “SNP within-group” model, (c) the “Hybrid genotype” model for the single-marker analysis. The chromosome number is indicated on the X-axis. For each model, graphics correspond to the test of the global effect (on the top) or of one component (the Flint GCA, the Dent GCA and the SCA for the “Founder alleles” and “SNP within group” models and the additive and dominance effect for the “Hybrid genotype” model). The blue dots correspond to positions that were above the threshold in the single-marker analysis (see File S3). The red squares correspond to the –log(p-value) of the QTLs that were included in the final multi-locus model, with tests conditioned by the other QTL effects of the final detection model. An upper limit for the –log(pval) was fixed to 16

**
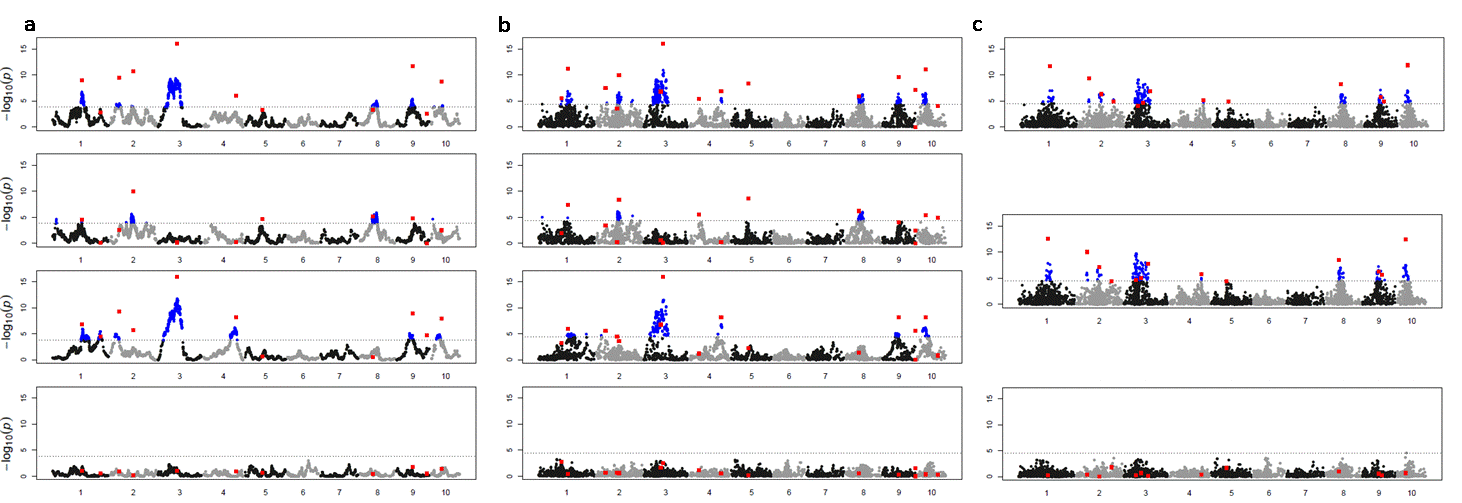
**

Founder Alleles

Global

FlintGCA

DentGCA

SCA

SCA

DentGCA

FlintGCA

Global

Hybrid genotype

SNP within group

Global

Additivity

Dominance

**Figure S3:** -log(p-value) curves of QTL detection for Plant height (PH) with (a) the “Founder alleles” model, (b) the “SNP within-group” model, (c) the “Hybrid genotype” model for the single-marker analysis. The chromosome number is indicated on the X-axis. For each model, graphics correspond to the test of the global effect (on the top) or of one component (the Flint GCA, the Dent GCA and the SCA for the “Founder alleles” and “SNP within group” models and the additive and dominance effect for the “Hybrid genotype” model). The blue dots correspond to positions that were above the threshold in the single-marker analysis (see File S3). The red squares correspond to the –log(p-value) of the QTLs that were included in the final multi-locus model, with tests conditioned by the other QTL effects of the final detection model. An upper limit for the –log(pval) was fixed to 16


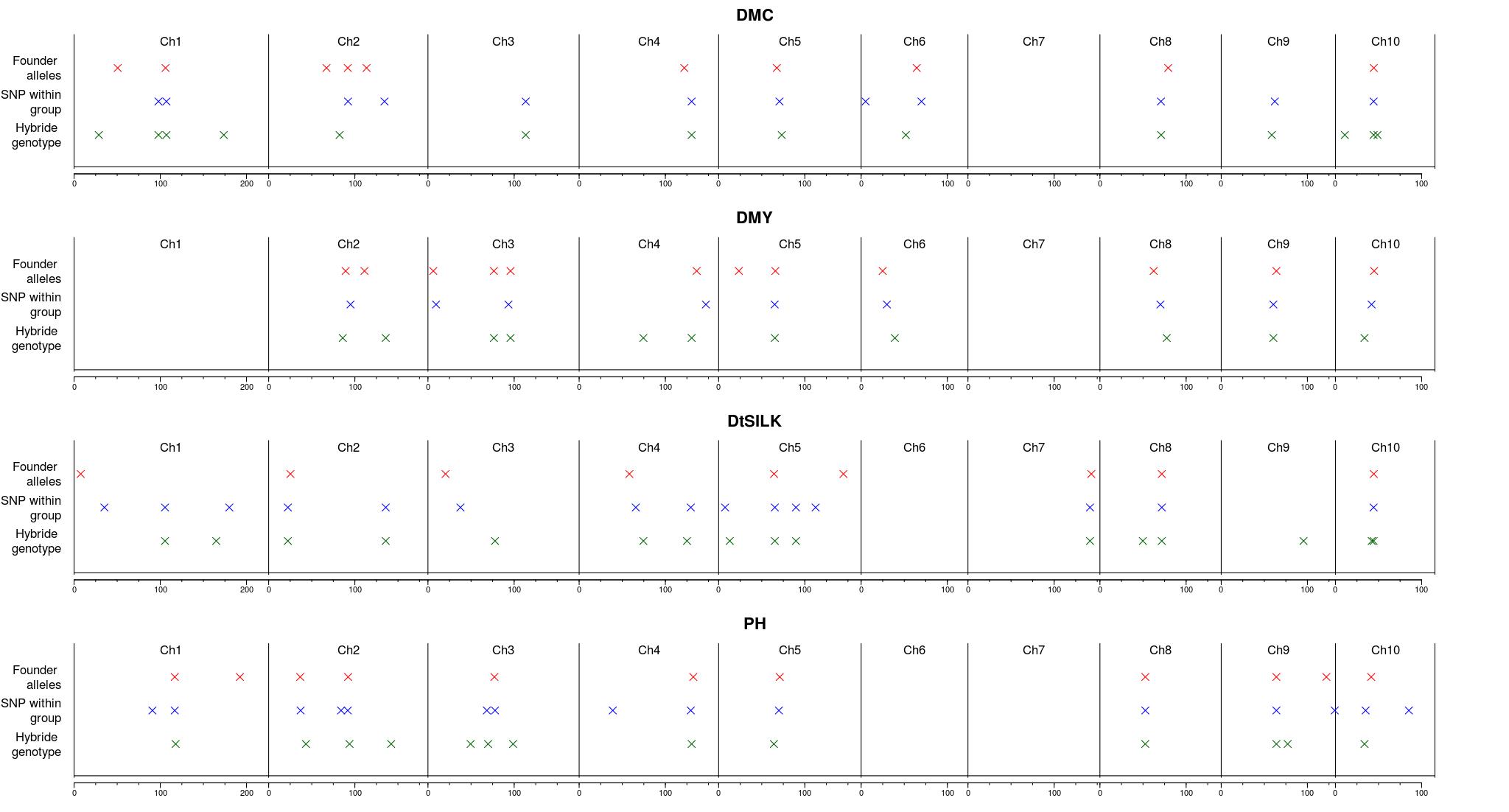


**Figure S4:** Synthesis of the positions of the QTLs detected for the four studied traits (dry matter content, DMC; dry matter yield, DMY; female flowering time DtSILK, and plant height, PH) and the different models. The QTLs detected with the “Founder alleles”, “SNP within-group” and “Hybrid genotype” models are indicated respectively with red, blue and green crosses.

**Table S1:** Individual population and consensus genetic maps: number (Nb) of mapped markers, number of unique genetic map positions, genetic map length, average and maximum distance between consecutive marker positions (with the chromosome where it is located).

| Population | Nb of mapped markers | Nb of unique positions | Map length  (cM) | Average distance  (cM) | Max distance  (cM) and chromosome |
| --- | --- | --- | --- | --- | --- |
| F1 | 3600 | 1328 | 1610.6 | 1.2 | 47.8 (chr 4) |
| F2 | 3289 | 1172 | 1569.7 | 1.4 | 94.1 (chr 4) |
| F3 | 2928 | 809 | 1315.2 | 1.6 | 30.9 (chr 2) |
| F4 | 4110 | 1501 | 1672.7 | 1.1 | 16.9 (chr 8) |
| F5 | 3947 | 1206 | 1525.3 | 1.3 | 20.9 (chr 10) |
| F6 | 4160 | 1322 | 1541.2 | 1.2 | 22.8 (chr 5) |
| D1 | 3580 | 872 | 1643.9 | 1.9 | 35.3 (chr 1) |
| D2 | 3577 | 1019 | 1565.8 | 1.6 | 23.2 (chr 10) |
| D3 | 3249 | 932 | 1511.4 | 1.6 | 37.7 (chr 10) |
| D4 | 3987 | 1062 | 1626.4 | 1.5 | 25.5 (chr 7) |
| D5 | 4235 | 1174 | 1639.9 | 1.4 | 13.3 (chr 10) |
| D6 | 4316 | 899 | 1502.8 | 1.7 | 23.7 (chr 10) |
| Consensus | 9548 | 5216 | 1578.6 | 0.3 | 4.3 (chr 7) |

**Table S2: Results of the QTLs detection for the “Hybrid Genotype” model for the four studied traits: Dry Matter Content (DMC), Dry Matter Yield (DMY), Silking Date (DtSILK), Plant Height (PH).** For each significant QTL (for its total effect or one of its components), the chromosome (Chr), the marker (Mk), the genetic position in cM (Gen pos), the physical position in kbp (Phys pos), the –log(p-value) of the additive part of its effect (-log(Ad)), the –log(p-value) of the dominance part of its effect (-log(Dom)), the –log(p-value) of its global effect (-log(Global)), the explained R² in % (R²) are indicated.

| Trait | Chr | Mk | Gen pos | Phys pos | -log(Ad) | -log(Dom) | -log(Global) | R² |
| --- | --- | --- | --- | --- | --- | --- | --- | --- |
| DMC |  |  |  |  |  |  |  |  |
|  | 1 | PZE-101023852 | 28.7 | 14032 | 5.2 | 0.04 | 4.46 | 0.91 |
|  | 1 | PZE-101107138 | 97.9 | 112147 | 5.6 | 0.17 | 4.83 | 0.98 |
|  | 1 | PZE-101141198 | 107.1 | 182293 | 14.5 | 0.49 | 13.68 | 2.76 |
|  | 1 | PZE-101210621 | 173.7 | 260145 | 6.2 | 1.17 | 6.10 | 1.23 |
|  | 2 | PZE-102080558 | 82.3 | 64362 | 8.6 | 0.36 | 7.83 | 1.60 |
|  | 3 | PZE-103142982 | 113.4 | 198521 | 6.1 | 0.45 | 5.47 | 1.24 |
|  | 4 | PZE-104129789 | 130.5 | 210477 | 10.1 | 0.08 | 9.17 | 1.87 |
|  | 5 | PZE-105100982 | 73.2 | 151631 | 6.6 | 0.77 | 6.19 | 1.10 |
|  | 6 | PZE-106068323 | 51.9 | 121253 | 6.0 | 0.49 | 5.38 | 1.09 |
|  | 8 | PZE-108075290 | 71 | 130926 | 10.9 | 0.60 | 10.22 | 2.09 |
|  | 9 | PZE-109038235 | 58.7 | 56424 | 5.1 | 0.59 | 4.62 | 0.93 |
|  | 10 | PZE-110007567 | 10.9 | 5875 | 5.1 | 0.17 | 4.34 | 0.87 |
|  | 10 | PZE-110049918 | 44.3 | 94001 | 37.7 | 1.18 | 37.2 | 7.47 |
|  | 10 | PZE-110060381 | 48.8 | 114656 | 12.1 | 1.71 | 12.32 | 2.48 |
|  |  |  |  |  |  |  |  |  |
| DMY |  |  |  |  |  |  |  |  |
|  | 2 | PZE-102096468 | 86 | 111010 | 7.9 | 1.28 | 7.860 | 2.21 |
|  | 2 | PZE-102160945 | 135.8 | 207038 | 5.0 | 0.04 | 4.251 | 1.20 |
|  | 3 | PZE-103108225 | 76.5 | 169072 | 8.4 | 1.90 | 8.876 | 2.48 |
|  | 3 | PZE-103125956 | 95.8 | 183577 | 5.3 | 0.09 | 4.522 | 1.28 |
|  | 4 | PZE-104078143 | 74.5 | 152444 | 7.4 | 0.16 | 6.553 | 1.90 |
|  | 4 | PZE-104129789 | 130.5 | 210477 | 5.6 | 0.36 | 4.988 | 1.44 |
|  | 5 | PZE-105077552 | 65.2 | 87115 | 4.8 | 1.94 | 5.430 | 1.49 |
|  | 6 | PZE-106055176 | 39.1 | 105598 | 5.1 | 0.19 | 4.404 | 1.25 |
|  | 8 | PZE-108088583 | 77.5 | 145907 | 11.9 | 1.70 | 12.163 | 3.46 |
|  | 9 | PZE-109052698 | 60.5 | 91043 | 4.9 | 0.32 | 4.252 | 1.18 |
|  | 10 | PZE-110020953 | 33.7 | 27697 | 10.6 | 0.02 | 9.647 | 2.75 |
|  |  |  |  |  |  |  |  |  |
| DtSILK |  |  |  |  |  |  |  |  |
|  | 1 | PZE-101138117 | 105.5 | 179183 | 4.8 | 2.90 | 6.306 | 1.53 |
|  | 1 | PZE-101199598 | 164.9 | 248737 | 4.8 | 0.60 | 4.335 | 1.05 |
|  | 2 | PZE-102018300 | 22.3 | 8782 | 8.0 | 1.16 | 7.881 | 1.91 |
|  | 2 | PZE-102160945 | 135.8 | 207038 | 8.6 | 0.27 | 7.799 | 1.92 |
|  | 3 | PZE-103109418 | 77.8 | 170117 | 6.0 | 0.13 | 5.237 | 1.27 |
|  | 4 | PZE-104078143 | 74.5 | 152444 | 10.0 | 1.39 | 10.009 | 2.45 |
|  | 4 | PZE-104117192 | 125.1 | 193348 | 5.3 | 0.30 | 4.654 | 1.14 |
|  | 5 | PZE-105012348 | 12.9 | 5384 | 9.0 | 0.24 | 8.134 | 1.98 |
|  | 5 | PZE-105077552 | 65.2 | 87115 | 11.0 | 0.00 | 10.042 | 2.44 |
|  | 5 | PZE-105117653 | 89.7 | 174358 | 6.4 | 0.14 | 5.583 | 1.36 |
|  | 7 | PZE-107132427 | 141.6 | 172725 | 7.8 | 0.17 | 6.930 | 1.67 |
|  | 8 | PZE-108028681 | 49.8 | 26352 | 4.9 | 1.10 | 4.784 | 1.18 |
|  | 8 | PZE-108077879 | 71.8 | 133563 | 12.5 | 1.94 | 12.954 | 3.10 |
|  | 9 | PZE-109098682 | 95.5 | 143862 | 5.2 | 0.12 | 4.494 | 1.09 |
|  | 10 | PZE-110046358 | 42.1 | 87170 | 5.6 | 0.53 | 5.040 | 1.24 |
|  | 10 | PZE-110049918 | 44.3 | 94001 | 50.6 | 0.73 | 49.7 | 12.01 |
|  |  |  |  |  |  |  |  |  |
| PH |  |  |  |  |  |  |  |  |
|  | 1 | PZE-101152239 | 117.8 | 195684 | 12.6 | 0.30 | 11.75 | 2.67 |
|  | 2 | PZE-102037297 | 43.3 | 17988 | 10.1 | 0.41 | 9.30 | 2.11 |
|  | 2 | PZE-102119036 | 93.8 | 160729 | 7.2 | 0.03 | 6.33 | 1.41 |
|  | 2 | PZE-102168063 | 142.1 | 211948 | 4.4 | 1.83 | 4.94 | 1.08 |
|  | 3 | PZE-103051361 | 49.5 | 56901 | 4.7 | 0.29 | 4.03 | 0.92 |
|  | 3 | PZE-103102119 | 69.8 | 162433 | 5.0 | 0.71 | 4.63 | 1.02 |
|  | 3 | PZE-103128864 | 98.8 | 185839 | 7.7 | 0.16 | 6.90 | 1.54 |
|  | 4 | PZE-104129789 | 130.5 | 210477 | 5.8 | 0.40 | 5.12 | 1.16 |
|  | 5 | PZE-105075570 | 64.1 | 83398 | 4.4 | 1.70 | 4.87 | 1.04 |
|  | 8 | PZE-108036758 | 52.6 | 57215 | 8.5 | 1.00 | 8.20 | 1.86 |
|  | 9 | PZE-109061773 | 64 | 103353 | 6.3 | 0.59 | 5.79 | 1.31 |
|  | 9 | PZE-109082918 | 77.1 | 131575 | 5.6 | 0.27 | 4.93 | 1.13 |
|  | 10 | PZE-110020737 | 33.7 | 27318 | 12.5 | 0.71 | 11.87 | 2.68 |

**Table S3: Results of the QTLs detection for the “SNP within-group” model for the four studied traits: Dry Matter Content (DMC), Dry Matter Yield (DMY), Silking Date (DtSILK), Plant Height (PH).** For each significant QTL (for its total effect or one of its components), the chromosome (Chr), the marker (Mk), the genetic position in cM (Gen pos), the physical position in kbp (Phys pos), the –log(p-value) of the flint GCA part of its effect (-log(GCAf)), the –log(p-value) of the dent GCA part of its effect (-log(GCAd) ), the –log(p-value) of the SCA part of its effect (-log(SCA)), the –log(p-value) of its global effect (-log(Global)), the explained R² in % (R²) are indicated.

| Trait | Chr | Mk | Gen pos | Phys pos | -log (GCAf) | -log (GCAd) | -log (SCA) | -log (Global) | R² |
| --- | --- | --- | --- | --- | --- | --- | --- | --- | --- |
| DMC |  |  |  |  |  |  |  |  |  |
|  | 1 | PZE-101107138 | 97.9 | 112147 | 4.01 | 3.63 | 0.17 | 6.31 | 1.42 |
|  | 1 | PZE-101141198 | 107.1 | 182293 | 6.72 | 5.61 | 0.15 | 9.73 | 2.15 |
|  | 2 | PZE-102116089 | 92.1 | 153797 | 1.86 | 6.73 | 0.91 | 7.04 | 1.58 |
|  | 2 | PZE-102159268 | 134.4 | 205898 | 0.11 | 7.26 | 2.14 | 7.29 | 1.64 |
|  | 3 | PZE-103142982 | 113.4 | 198521 | 4.95 | 2.35 | 0.67 | 5.70 | 1.30 |
|  | 4 | PZE-104129789 | 130.5 | 210477 | 6.22 | 4.59 | 0.35 | 8.68 | 1.94 |
|  | 5 | PZE-105096988 | 70.6 | 142855 | 5.26 | 1.26 | 0.72 | 5.10 | 1.17 |
|  | 6 | PZE-106006210 | 5.2 | 7781 | 0.27 | 4.84 | 0.01 | 3.56 | 0.84 |
|  | 6 | PZE-106090096 | 70 | 147212 | 7.53 | 6.71 | 1.40 | 12.01 | 2.62 |
|  | 8 | PZE-108074836 | 70.8 | 130409 | 1.76 | 8.92 | 0.36 | 8.99 | 1.98 |
|  | 9 | PZE-109057266 | 62.2 | 98436 | 4.64 | 1.15 | 0.23 | 4.07 | 0.95 |
|  | 10 | PZE-110049918 | 44.3 | 94001 | 38.78 | 5.18 | 0.02 | 41.13 | 8.64 |
| DMY |  |  |  |  |  |  |  |  |  |
|  | 2 | PZE-102120732 | 95 | 164884 | 8.96 | 3.59 | 0.10 | 9.81 | 2.95 |
|  | 3 | PZE-103008521 | 9.4 | 4704 | 0.82 | 4.71 | 0.49 | 3.83 | 1.29 |
|  | 3 | PZE-103124449 | 93.4 | 181769 | 0.71 | 12.18 | 0.17 | 10.66 | 3.33 |
|  | 4 | PZE-104144717 | 147 | 233539 | 2.73 | 3.59 | 0.50 | 4.64 | 1.50 |
|  | 5 | PZE-105077135 | 65 | 86222 | 5.23 | 3.42 | 0.14 | 6.77 | 2.10 |
|  | 6 | PZE-106048775 | 29.9 | 97805 | 0.24 | 5.03 | 0.40 | 3.92 | 1.33 |
|  | 8 | PZE-108073565 | 70.2 | 128542 | 3.69 | 12.84 | 0.81 | 14.40 | 4.42 |
|  | 9 | PZE-109052698 | 60.5 | 91043 | 4.55 | 0.60 | 0.32 | 3.61 | 1.16 |
|  | 10 | PZE-110045871 | 41.9 | 86698 | 5.27 | 7.23 | 0.02 | 10.20 | 3.16 |
| DtSILK |  |  |  |  |  |  |  |  |  |
|  | 1 | PZE-101029748 | 35.2 | 17832 | 0.65 | 6.54 | 0.80 | 5.93 | 1.52 |
|  | 1 | PZE-101138117 | 105.5 | 179183 | 7.02 | 0.30 | 0.70 | 5.99 | 1.56 |
|  | 1 | PZE-101217474 | 180.1 | 268814 | 5.26 | 2.41 | 0.07 | 5.69 | 1.50 |
|  | 2 | PZE-102018300 | 22.3 | 8782 | 1.56 | 8.25 | 0.63 | 8.38 | 2.11 |
|  | 2 | PZE-102160945 | 135.8 | 207038 | 5.20 | 4.35 | 0.24 | 7.80 | 2.00 |
|  | 3 | PZE-103026244 | 37.7 | 19256 | 1.02 | 6.71 | 0.50 | 5.82 | 1.50 |
|  | 4 | PZE-104058608 | 65.7 | 112760 | 0.60 | 8.22 | 0.22 | 6.87 | 1.76 |
|  | 4 | PZE-104126472 | 129.5 | 204417 | 3.38 | 3.72 | 0.13 | 5.02 | 1.33 |
|  | 5 | PZE-105006205 | 7.5 | 3143 | 3.68 | 6.81 | 0.06 | 8.02 | 2.01 |
|  | 5 | PZE-105077552 | 65.2 | 87115 | 6.56 | 5.09 | 0.21 | 9.82 | 2.47 |
|  | 5 | PZE-105117617 | 89.7 | 174351 | 0.34 | 6.13 | 0.13 | 4.85 | 1.27 |
|  | 5 | PZE-105138874 | 112.5 | 193728 | 0.00 | 4.94 | 0.48 | 3.82 | 1.01 |
|  | 7 | PZE-107132427 | 141.6 | 172725 | 9.88 | 0.83 | 0.53 | 8.85 | 2.23 |
|  | 8 | PZE-108077879 | 71.8 | 133563 | 0.29 | 19.40 | 0.57 | 17.84 | 4.30 |
|  | 10 | PZE-110049918 | 44.3 | 94001 | 47.23 | 12.82 | 0.69 | 57.53 | 13.72 |
|  |  |  |  |  |  |  |  |  |  |
| PH |  |  |  |  |  |  |  |  |  |
|  | 1 | PZE-101091535 | 90.9 | 83644 | 1.94 | 3.27 | 2.81 | 5.50 | 1.15 |
|  | 1 | PZE-101150835 | 116.8 | 194674 | 7.42 | 5.91 | 0.45 | 11.24 | 2.39 |
|  | 2 | PZE-102032234 | 37.1 | 15025 | 3.48 | 5.64 | 0.71 | 7.48 | 1.62 |
|  | 2 | PZE_102084168 | 84 | 71896 | 0.24 | 4.44 | 0.65 | 3.50 | 0.79 |
|  | 2 | PZE-102115483 | 91.8 | 152503 | 8.42 | 3.61 | 0.61 | 10.02 | 2.15 |
|  | 3 | PZE-103100427 | 68.3 | 160672 | 0.67 | 6.75 | 1.67 | 6.81 | 1.44 |
|  | 3 | PZE-103109418 | 77.8 | 170117 | 0.12 | 27.23 | 2.48 | 27.10 | 5.31 |
|  | 4 | PZE-104019121 | 39 | 19433 | 5.50 | 1.21 | 1.20 | 5.43 | 1.22 |
|  | 4 | PZE-104126472 | 129.5 | 204417 | 0.28 | 8.15 | 0.53 | 6.89 | 1.45 |
|  | 5 | PZE-105094920 | 69.9 | 139235 | 8.63 | 2.20 | 0.17 | 8.35 | 1.83 |
|  | 8 | PZE-108038507 | 52.7 | 62847 | 6.24 | 1.41 | 0.60 | 5.89 | 1.33 |
|  | 9 | PZE-109061773 | 64 | 103353 | 4.08 | 8.17 | 0.33 | 9.59 | 2.06 |
|  | 9 | PZE-109121844 | 131.7 | 155733 | 2.41 | 5.59 | 1.57 | 7.10 | 1.54 |
|  | 10 | PZE_110035195 | 35 | 66734 | 5.40 | 8.20 | 0.37 | 11.07 | 2.36 |
|  | 10 | PZE-110094832 | 85.2 | 142069 | 4.90 | 0.83 | 0.34 | 4.09 | 0.94 |

**Table S4: Results of the QTLs detection for the “Founder Alleles” model for the four studied traits: Dry Matter Content (DMC), Dry Matter Yield (DMY), Silking Date (DtSILK), Plant Height (PH).** For each significant QTL (for its total effect or one of its components), the chromosome (Chr), the marker (Mk), the genetic position in cM (Gen pos), the physical position in kbp (Phys pos), the –log(p-value) of the flint GCA part of its effect (-log(GCAf)), the –log(p-value) of the dent GCA part of its effect (-log(GCAd)), the –log(p-value) of the SCA part of its effect (-log(SCA)), the –log(p-value) of its global effect (-log(Global)), the explained R² in % (R²) are indicated.

| Trait | Chr | Mk | Gen pos | Phys pos | -log (GCAf) | -log (GCAd) | -log  (SCA) | -log  (Global) | R² |
| --- | --- | --- | --- | --- | --- | --- | --- | --- | --- |
| DMC | 1 | PZE_101043600 | 50.6 | 29682 | 4.29 | 2.23 | 1.38 | 5.48 | 2.05 |
|  | 1 | PZE_101139638 | 106.1 | 180401 | 10.32 | 5.55 | 0.19 | 11.37 | 3.35 |
|  | 2 | PZE_102057464 | 67.1 | 36146 | 1.21 | 5.28 | 0.29 | 3.84 | 1.67 |
|  | 2 | PZE_102115483 | 91.8 | 152503 | 2.48 | 4.54 | 3.26 | 7.95 | 2.60 |
|  | 2 | PZE_102139190 | 113.6 | 187622 | 3.23 | 5.65 | 1.65 | 7.53 | 2.50 |
|  | 4 | PZE_104114190 | 122 | 189586 | 3.42 | 4.90 | 0.41 | 5.49 | 2.06 |
|  | 5 | PZE_105087042 | 67.5 | 111541 | 4.20 | 1.32 | 0.75 | 3.86 | 1.67 |
|  | 6 | PZE_106084109 | 64.5 | 141285 | 5.47 | 5.90 | 0.56 | 7.70 | 2.55 |
|  | 8 | PZE_108090858 | 79.2 | 148086 | 0.36 | 8.12 | 2.46 | 8.17 | 2.62 |
|  | 10 | PZE_110050269 | 44.5 | 94965 | 33.4 | 6.98 | 0.46 | 34.5 | 7.91 |
|  |  |  |  |  |  |  |  |  |  |
| DMY |  |  |  |  |  |  |  |  |  |
|  | 2 | PZE_102109928 | 89.3 | 141598 | 4.34 | 0.74 | 1.73 | 4.56 | 2.25 |
|  | 2 | PZE_102136109 | 111.2 | 185694 | 5.36 | 0.22 | 2.37 | 5.52 | 2.55 |
|  | 3 | PZE_103005573 | 6.2 | 3433 | 0.01 | 5.02 | 1.97 | 4.57 | 2.27 |
|  | 3 | PZE_103108225 | 76.5 | 169072 | 2.36 | 1.80 | 2.74 | 4.93 | 2.33 |
|  | 3 | PZE_103125892 | 95.8 | 183555 | 1.20 | 5.65 | 0.08 | 3.31 | 1.92 |
|  | 4 | PZE_104138099 | 136.3 | 225269 | 1.83 | 4.47 | 1.95 | 5.86 | 2.66 |
|  | 5 | PZE_105020816 | 23.5 | 10009 | 4.59 | 0.62 | 0.62 | 3.47 | 1.90 |
|  | 5 | PZE_105078900 | 65.8 | 89718 | 5.93 | 2.61 | 0.41 | 5.69 | 2.57 |
|  | 6 | PZE_106044414 | 25 | 93501 | 0.06 | 5.80 | 0.35 | 3.26 | 1.90 |
|  | 8 | PZE_108062087 | 62.4 | 110990 | 3.25 | 12.31 | 0.96 | 12.10 | 4.39 |
|  | 9 | PZE_109061773 | 64 | 103353 | 6.12 | 0.20 | 0.96 | 4.47 | 2.24 |
|  | 10 | PZE_110051444 | 44.9 | 96836 | 2.86 | 4.53 | 0.42 | 4.93 | 2.39 |
|  |  |  |  |  |  |  |  |  |  |
| DtSILK |  |  |  |  |  |  |  |  |  |
|  | 1 | PZE-101004387 | 7.7 | 3883 | 4.37 | 2.28 | 0.56 | 4.37 | 2.40 |
|  | 2 | PZE-102021400 | 25.3 | 9973 | 1.09 | 4.05 | 0.96 | 3.77 | 2.22 |
|  | 3 | PZE-103016459 | 20.4 | 9311 | 1.26 | 5.30 | 0.10 | 3.48 | 2.13 |
|  | 4 | PZE-104034811 | 58.3 | 43970 | 2.02 | 6.75 | 0.74 | 6.02 | 2.98 |
|  | 5 | PZE-105075954 | 64.3 | 83980 | 10.43 | 2.84 | 0.61 | 10.16 | 4.16 |
|  | 5 | PZE-105166980 | 144.8 | 210286 | 0.47 | 3.32 | 3.68 | 5.51 | 2.71 |
|  | 7 | PZE-107133704 | 143.1 | 173181 | 5.53 | 1.46 | 2.87 | 7.32 | 3.23 |
|  | 8 | PZE-108077879 | 71.8 | 133563 | 0.39 | 11.26 | 0.54 | 8.21 | 3.63 |
|  | 10 | PZE-110050273 | 44.5 | 94968 | 32.5 | 11.90 | 0.83 | 39.5 | 11.68 |
|  |  |  |  |  |  |  |  |  |  |
| PH |  |  |  |  |  |  |  |  |  |
|  | 1 | PZE_101150835 | 116.8 | 194674 | 4.54 | 6.87 | 1.01 | 9.01 | 2.88 |
|  | 1 | PZE_101233132 | 192.4 | 281321 | 0.13 | 4.42 | 0.57 | 2.79 | 1.42 |
|  | 2 | PZE_102031753 | 36.6 | 14800 | 2.56 | 9.27 | 0.90 | 9.42 | 3.03 |
|  | 2 | PZE_102116344 | 92.2 | 154273 | 9.96 | 5.75 | 0.20 | 10.67 | 3.32 |
|  | 3 | PZE_103108648 | 77.1 | 169577 | 0.19 | 20.8 | 1.04 | 17.1 | 4.42 |
|  | 4 | PZE_104134644 | 132.4 | 218941 | 0.24 | 8.16 | 0.94 | 6.05 | 2.17 |
|  | 5 | PZE_105097751 | 70.9 | 144099 | 4.71 | 0.61 | 0.62 | 3.34 | 1.57 |
|  | 8 | PZE_108038053 | 52.7 | 61790 | 5.22 | 0.50 | 0.41 | 3.29 | 1.59 |
|  | 9 | PZE_109061773 | 64 | 103353 | 4.85 | 8.94 | 1.74 | 11.71 | 3.49 |
|  | 9 | PZE_109115897 | 122 | 152795 | 0.01 | 4.68 | 0.50 | 2.59 | 1.34 |
|  | 10 | PZE_110045521 | 41.5 | 85840 | 2.52 | 7.94 | 1.41 | 8.74 | 2.80 |
